# The Medical College of Ohio

**Published:** 1837

**Authors:** 


### Art. V.—The Medical College of Ohio.

This ill-fated institution has lately suffered the loss of half its faculty; Professors Cross, Smith and Eberle having resigned, and removed from Cincinnati. D.

